# Method for Automatic Detection of the Technical State of Insulated Coolboxes in the Cold Chain Based on IoT Sensor Data

**DOI:** 10.3390/ma19204306

**Published:** 2026-10-13

**Authors:** Augustyn Lorenc, Małgorzata Kuźnar, Janusz Pobędza, Artur Gawlik, Artur Guzowski

**Affiliations:** 1 Department of Transport and Logistics Systems (L-6), Faculty of Civil Engineering and Transport, PK Krakow University of Technology, 31-878 Krakow, Poland; 2 Department of Rail Vehicles and Transport, Faculty of Mechanical Engineering, PK Krakow University of Technology, 31-864 Krakow, Poland; malgorzata.kuznar@pk.edu.pl; 3 Laboratory of Techno-Climatic Research and Heavy Duty Machines, Faculty of Mechanical Engineering, PK Krakow University of Technology, 31-864 Krakow, Poland; janusz.pobedza@pk.edu.pl (J.P.); artur.gawlik@pk.edu.pl (A.G.); artur.guzowski@pk.edu.pl (A.G.)

**Keywords:** cold chain, coolbox, eutectic plates, IoT sensors, thermal insulation, technical state diagnostics, predictive maintenance, food safety, insulated container

## Abstract

Maintaining temperature stability within the cold chain is critical to preventing food spoilage and financial loss. Insulated containers (coolboxes) using eutectic plates are widely employed to maintain passive cooling during transport. However, mechanical wear, seal damage, or improper latching can lead to microclimatic fluctuations inside the container, compromising product safety. This paper presents a non-invasive method for the automatic detection and classification of the technical state of coolboxes (fully functional, conditionally functional, and faulty) based on internal temperature and humidity measurements collected via wireless IoT sensors. Experimental trials conducted in a controlled climatic chamber simulating real-world operations demonstrate that micro-temperature gradients and heating rates allow for the early detection of seal degradation and incomplete door closures before critical thermal thresholds are violated. Specifically, the results confirm that uncompressed or damaged seals accelerate internal heating rates by up to 60%, and the diagnostic algorithm successfully classifies technical states with high sensitivity based on a degradation index threshold (∆*Q* ≤ 10% for fully functional, 10% < ∆*Q* ≤ 30% for conditionally functional, and ∆*Q* > 30% for faulty units). The proposed diagnostic framework supports predictive maintenance and improves overall cold-chain traceability.

## 1. Introduction

Maintaining strict temperature stability throughout cold chain logistics is a critical prerequisite for ensuring food safety, preserving product quality, and preventing catastrophic economic losses within the global food supply chain. Temperature-sensitive perishable goods—such as fresh meat, dairy, fruits, and pharmaceuticals—require uninterrupted thermal protection from the point of origin through processing and distribution centers (DCs) down to the final retail outlet. Passive thermal insulation containers, commonly referred to as coolboxes, integrated with eutectic phase-change materials (eutectic plates or cold accumulators), represent one of the most widely adopted and energy-efficient solutions for short- to medium-distance refrigerated transport. These autonomous passive cooling units eliminate the need for active refrigeration units inside delivery vehicles, substantially reducing fuel consumption and greenhouse gas emissions. However, repeated operational cycles expose coolboxes to aggressive mechanical stresses, harsh environmental conditions, and intensive cleaning procedures. Over time, these factors induce structural degradation, primarily manifested as the microstructural breakdown of Polyurethane (PUR) insulation foam, blowing gas diffusion, mechanical deformation of Polyethylene (PE) outer walls, and the progressive wear or loss of elasticity of rubber sealing gaskets. Furthermore, human errors during busy distribution operations—such as incomplete door closures or failure to fully engage locking levers—introduce severe thermal breaches. These physical defects create localized thermal bridges that drastically accelerate heat infiltration, compromising the passive cooling window and leading to undetected food spoilage before the expiration date. Traditionally, evaluating the physical aging and thermal insulation degradation of coolboxes relies on standard laboratory testing methods (such as extracting core wall samples to measure thermal conductivity via heat flow meter apparatuses in accordance with EN 12667:2001 [1] or ISO 8301:1991 [2] standards). Although accurate, these destructive testing procedures are exceptionally expensive, time-consuming, labor-intensive, and cause irreversible damage to the container, forcing its permanent removal from the operational fleet. Conversely, non-destructive quality control in logistics is largely restricted to manual visual inspections, which fail to quantify internal insulation degradation or micro-leakages along the sealing perimeter.

### State of the Art

Every year, the food sector—spanning both manufacturing enterprises and retail outlets—faces heavy financial burdens caused by goods spoiling prior to their expiry dates. These losses extend beyond mere monetary value, potentially damaging a company’s corporate image, particularly when partnering with major retail networks that enforce stringent quality protocols. Furthermore, producers fulfilling contracts for large-scale clients frequently lack complete oversight of the entire logistics chain. This discrepancy often arises because shipping operations are managed by the buyers themselves or by external logistics providers—i.e., third-party logistics (3PL).

The development of digital and sensor technologies is irreversibly transforming the modern food safety system, replacing traditional, reactive control methods with continuous, predictive real-time monitoring [3]. The implementation of smart packaging (featuring biosensors and RFID tags), Big Data analytics, and artificial intelligence algorithms enables precise product freshness assessment, quality inspection automation, and improved transparency across the entire supply chain [3]. However, the effective processing of complex, streaming data gathered from multiple distributed environmental sources remains a key challenge in this field [3,4].

Addressing these limitations requires innovative machine learning architectures targeted at multimodal data fusion and transport condition optimization [4,5]. An example of such an approach is a fruit and vegetable freshness detection system utilizing the Co-ACRN model, which integrates digital imagery with time series of gas and climate parameters by combining co-attention and self-attention mechanisms with CNN and LSTM networks, achieving a detection accuracy of 96.93% in under 2 s [4]. Meanwhile, in cold chain logistics, applying the TD3++ reinforcement learning algorithm integrated with IoT sensor networks (GPS, thermal imaging cameras, and accelerometers) enabled dynamic optimization of routes and transport parameters, reducing cold chain violations by 53% and transport costs by 23% [5].

Ensuring the continuity and stability of environmental conditions in the cold chain is of fundamental importance for preserving the quality and safety of delicate goods, such as food and pharmaceutical products [6]. This process encompasses the full cycle, including processing, storage, transport, and distribution, which generates significant risk of loss given the logistical complexity [6]. To mitigate this risk, continuous monitoring of basic parameters—primarily temperature and humidity—and, in fruit ripening processes (e.g., apples), gas concentrations such as ethylene, becomes key [7]. Integrating wireless sensors (e.g., LM35, DHT11, DS18B20, or MQ-135 gas sensors) with microcontrollers such as Arduino or ESP32 enables continuous, real-time data acquisition [8,9,10,11].

Communication and data transmission in IoT systems are implemented using diverse wireless technologies. The Bluetooth standard and its low-energy version—i.e., Bluetooth Low Energy (BLE)—allow for efficient and direct data reading from tracking devices, simultaneously guaranteeing long battery life and smooth communication using mobile devices [12,13]. In infrastructures requiring a direct cloud connection, Wi-Fi modules and data transfer protocols such as MQTT are widely used; thanks to its lightweight nature, MQTT performs well under unstable network connection conditions [8,9,14]. Combining environmental parameter sensors with GPS modules allows not only for supervising cold room or container temperatures, but also for real-time verification of the transport’s geographical location [11,15].

The application of IoT solutions in warehousing and distribution translates into improved operational processes and reduced unnecessary human labor. Smart warehouse systems can integrate vision cameras for object identification and weight sensors (e.g., load cells with the HX711 chip and Raspberry Pi) for automated inventory tracking, notifying users of potential stock shortages [10]. Through real-time alerts and dashboards (IoT Dashboards), stakeholders participating in the supply chain gain complete transparency, enabling an immediate response in emergency situations—such as during the transport of vaccines or sensitive medical supplies—preventing damage to the cargo [7,8,9,15].

Operational management in cold chain logistics is subject to numerous critical risk factors that can lead to system-wide failure. Studies using Bayesian networks show that the stage of the cold chain most vulnerable to failure is transport and distribution, while transport equipment breakdowns, extreme weather conditions, improper pre-cooling, and handling errors are cited among the key events threatening delivery continuity [16]. IoT monitoring systems allow for effective mitigation of these types of threats by increasing temperature stability and shortening response times to logistical disruptions [9].

To ensure the reliability of refrigeration equipment and complex industrial systems, predictive maintenance (PdM) implemented within Industry 4.0 and Cyber-Physical Systems is gaining increasing importance [17,18]. Although purely data-driven approaches achieve high predictive performance, they are characterized by limitations in knowledge generalization and uncertainty estimation [17,18].

The solution to these issues lies in deploying hybrid models that combine deep learning with physics and domain expertise. An example is the integration of Bayesian Neural Network (BNN) models with physical models in variable refrigerant flow (VRF) systems, which significantly reduces epistemological uncertainty and yields better physical interpretability of results [19]. In turn, applying hybrid networks (e.g., physically informed CNN-LSTM or DK-LSTM architectures) in combination with Digital Twins enables precise forecasting of the remaining useful life (RUL) of equipment [17,20]. Such a hybrid approach exhibits high robustness against missing sensor data, domain shifts, and noise, constituting a key element of safety in critical industrial and marine applications [17,20].

Synthesizing these achievements, advanced analytics based on sensor fusion and AI algorithms are becoming the foundation of modern food quality management [3,4,5]. The synergy of intelligent edge detection optimized for fast inference [4] with advanced predictive analytics at the logistical network level [5] not only minimizes food loss and health risks but also tangibly reduces carbon dioxide emissions [3,5]. Further advancement in this field will require increasing hardware scalability, reducing deployment costs for advanced sensor arrays, and ensuring system interoperability across global supply chains [3,5].

To mitigate temperature excursion risks, recent advances in IoT (Internet of Things) technologies and sensor miniaturization have enabled continuous microclimatic monitoring during transit. Modern cold chain management systems utilize wireless sensor networks (WSNs), RFID tags, and cloud-based analytical platforms to record internal temperature and humidity profiles. However, existing commercial systems and research solutions suffer from critical operational limitations:

- Vulnerability of Dual-Sensor Architectures: Conventional thermal leakage detection methods rely on mounting temperature sensors on both the interior and exterior surfaces of each container to compute real-time thermal gradients. In industrial logistics, external sensors mounted on container outer walls are highly susceptible to severe mechanical impacts during forklift handling, physical abrasion during transit, theft, and thermal destruction during high-pressure container washing cycles executed at high temperatures.
- Trailer-Centric Blind Spots: Standard transport monitoring typically tracks ambient climate conditions at the vehicle or semi-trailer level rather than inside individual passive containers. Because airflow and cooling inside a refrigerated semi-trailer are inherently uneven, ambient trailer data fails to detect localized insulation failures or unlatched doors on individual coolboxes.
- Reactive vs. Predictive Capabilities: Most commercial telemetry solutions generate alarms only after internal temperatures violate fixed regulatory thresholds (e.g., ATP standards for fresh produce). By the time an absolute thermal threshold is breached, product quality has already been compromised. Existing approaches lack predictive mechanisms capable of identifying progressive material decay before a critical failure occurs.

Maintaining the cold chain relies heavily on tracking thermal and moisture fluctuations inside transport vehicles and administering environmental data logs. A multitude of market-ready hardware systems, software applications, and technology vendors currently provide diverse tools designed for capturing, archiving, and accessing this information.

The evaluation of food safety during transit has attracted significant academic attention, as explored by authors such as Martins et al. [21], Hopper et al. [22], Fangchun et al. [23], Vinodini Ramesh and Narayanadas [24], and Xiang and Zhang [25]. Zhang et al. [26] introduced an IoT-based smart monitoring framework capable of supervising climate parameters within refrigerated vehicles, performing automated cargo recognition, and tracking real-time vehicle positioning throughout the shipping cycle using RFID and sensor equipment [27].

Ryan [28] identified fifteen primary risk areas associated with food transport, encompassing refrigeration and temperature regulation, transport unit administration, packaging, loading and unloading, security, pest management, container design, preventive maintenance, personnel hygiene, policy frameworks, the handling of rejected shipments, holding storage, and traceability. Regardless of the specific food items being hauled, every product undergoes a series of shared stages along its path from origin to point of use to guarantee safe transit and prevent contamination. Modern trends in food safety concentrate on downsizing analytical procedures using sensors, biosensors, microchips, lab-on-a-chip technology, or micro total analysis systems, which allow for the swift identification of potential contaminants, particularly during transport. These compact devices measure various metrics, including temperature, humidity, chemical agents, and microbial pollutants [29].

Although existing commercial technologies and academic studies enable the tracking of environmental parameters inside cargo trailers, they fail to capture the actual microclimate surrounding the products themselves. Uneven airflow frequently creates thermal discrepancies across the semi-trailer, typically producing colder zones near the front and elevated temperatures in the central section. Consequently, positioning data loggers incorrectly yields erroneous measurements. Furthermore, shipments often undergo drastic thermal swings while waiting for unloading or transitioning between transport modes (such as shifting cargo from freight trucks to aircraft). Independent industry analyses indicate that temperature differentials can reach as high as 75 °C during these transition phases.

Even though spikes in thermal and moisture levels do not pinpoint a specific category of contamination, they act as critical warning signs for potential degradation across diverse food commodities, including dairy, meat, fresh produce, fruits, and vegetables. For this reason, temperature and humidity function as versatile, universal metrics for engineering monitoring sensors applicable to a broad spectrum of foodstuffs [30].

To address these challenges, this paper proposes a novel, data-driven, non-destructive diagnostic methodology for the continuous assessment of technical state decay and insulation integrity in passive coolboxes.

The core contribution of this work lies in an algorithm that evaluates thermal insulation quality (Thermal Integrity Index—*I_TI_*) and detects progressive decay (*ΔQ*) using only a single internal IoT temperature sensor. Instead of requiring dedicated external hardware on each container wall, the algorithm dynamically retrieves ambient environmental data (*T_ext_weather_*) from location-based weather APIs using approximate GPS coordinates provided by the fleet’s Transport Management System (TMS).

By leveraging operational repeatability–specifically, comparing internal heating rates ($\frac{\mathrm{dT}}{\mathrm{dt}}$) across recurring transport loops under similar cargo fill volumes (*V_load_*), cargo heat capacities (*C_cargo_*), and environmental profiles—the proposed method achieves the following objectives:

- Eliminates Destructive Testing: Enables continuous, inline tracking of PUR foam aging and seal deterioration throughout the asset’s lifecycle without destroying or taking the container out of service.
- Reduces Hardware Costs and Failures: Removes all external sensor requirements, eliminating sensor damage risks during automated high-temperature container washing and heavy warehouse handling.
- Enables Predictive Maintenance: Differentiates between immediate operational faults (e.g., unlatched door levers or open door gaps) and long-term material degradation, allowing logistics operators to transition from reactive repairs to condition-based seal replacement and maintenance.

The performance and diagnostic sensitivity of the proposed methodology were empirically validated through full-scale experimental trials conducted in an accredited walk-in thermoclimatic chamber at the Laboratory of Techno-Climatic Research and Heavy Machinery (LBT) under simulated real-world cold-chain operations.

## 2. Materials and Methods

### 2.1. Coolbox Material

Insulated containers (coolboxes) represent passive refrigeration solutions designed for the safe and efficient transport of temperature-controlled products (fresh, frozen, or industrial/pharmaceutical).

Fully reusable, easy to wash and disinfect, and equipped with replaceable cooling inserts (eutectic plates/ice packs), the container acts as a self-sustaining cold source. This system enables stable temperature maintenance for 24 h or longer, protecting cargo quality across the entire logistical chain—from the warehouse to the final destination.

ATP certification confirms the highest quality of thermal insulation and approves the container for journeys exceeding 80 km, accounting for so-called load breaks (freight transfers), during which the main transport cargo space is opened.

Design and Materials

- Thermal Insulation (Core): Polyurethane Foam (PUR): Seamlessly foamed, high-density foam free of CFCs and HCFCs. Guarantees an excellent heat transfer coefficient (K ≈ 0.33 W/m^2^∙K) and protection against energy losses.
- Walls and Casing: Food-Grade Polyethylene (PE): Rotomolded double-walled body without seams. Impact-resistant, UV-stabilized, and resistant to mechanical corrosion as well as aggressive chemicals used during pressure washing and disinfection.
- Connecting Elements and Fasteners:
  ◦ Steel: Structural reinforcements, fittings, hinges, and a latch recessed into the door profile made of steel. Ensures high wear resistance under heavy warehouse and transport operations.
  ◦ Rubber sealing: Perimeter gaskets made of high-quality, low-temperature-resistant elastomer. Ensures hermetic sealing of the internal chamber, preventing ambient warm air ingress.
- Cold Source (Coolant/Eutectic System): In accordance with manufacturer technological standards (producer: Olivo/Melform):
  ◦ Natural Convective Cooling: The system utilizes eutectic plates or cold accumulators mounted on the ceiling or upper brackets of the container. These plates act as passive thermal sinks rather than active cooling devices. As the air in immediate contact with the cold plates cools down, its density increases. Due to gravity, this cooler, denser air naturally descends toward the bottom of the container, displacing warmer air upwards. This creates a passive natural (gravitational) convection loop, ensuring temperature distribution across the entire 768 L volume, as opposed to forced-air convection driven by mechanical fans.
  ◦ Eutectic Fluid Selection: Formulated based on the required transport temperature range:
    ▪ Fresh Products: 0 °C to +4 °C
    ▪ Frozen Products: Down to −21 °C
    ▪ Deep Frozen/Dry Ice: Below −25 °C

Table 1 shows the materials of the isothermal container and their properties.

**Table 1:** Thermal properties of materials used in coolboxes [31].

Material	Density [kg/m^3^]	Thermal Conductivity [W/mK]	Specific Heat Capacity [J/kgK]
polyethylene (PE)	911	0.461	1810.0
polyurethane foam (PUR)	43.2	0.0255	1280.0
steel	8055.0	15.1	480.0
rubber	1100.0	0.9	692.0

The properties of the coolboxes used in research:

- Outer dim. in mm (L × D × H): 800 × 1000 × 1900 mm
- Inner dim. in mm (L × D × H): 610 × 820 × 1535 mm
- Inner volume: 768 Liters
- Unloaded weight: 130 Kg
- Insulating material: Polyurethane foam (PUR)
- Wall material: Food-grade polyethylene (PE)
- Connecting elements, hinges, lock: Steel
- Seal: Rubber

### 2.2. Logistics Process

The management of containers in the process depends on each client. There are no requirements specifying how such a process should look, which is why the process may vary for each client. Similarly, the transportation temperature and the temperature in individual zones within the distribution center depend on the client. Maintaining stringent thermal conditions across perishable supply chains is not only critical for food safety and waste reduction, but it also dictates the overall energy efficiency of logistical operations. Inefficient thermal insulation and unoptimized transport packaging lead to excessive energy consumption during preliminary cooling stages and increase the carbon footprint of distribution networks [32,33]. The processes involved in the container cycle are presented in Figure 1.

**Figure 1:**
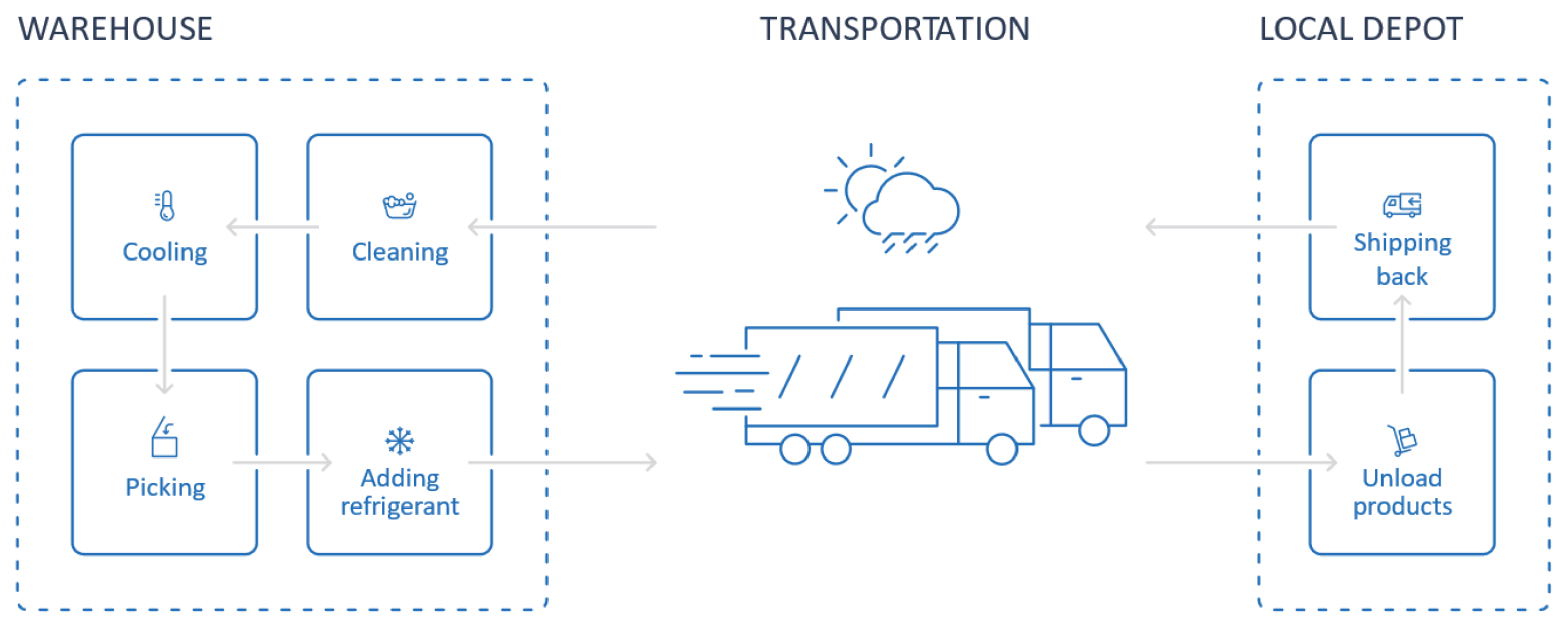
General flowchart of the logistics cycle.

Table 2 presents a detailed breakdown of the logistics cycle divided into warehouse zones and the requirements for the execution of each individual process.

In the logistics cycle, the container washing and cooling process is of particular importance. These processes do not have to occur in every cycle, as this depends on the internal policy of the company. In some cases, container washing takes place before the start of each cycle; in others, it occurs periodically—e.g., once every few cycles or on a specific day of the week—and some companies wash containers only when dirt is visible or when an unpleasant odor is detected inside.

Container drying can be performed in two ways: drying in dedicated rooms with forced air circulation at a higher temperature than in the rest of the distribution center (DC), or in a designated zone without elevated temperatures or forced air circulation.

### 2.3. Method of Automatically Detecting the Technical State of the Coolbox

The algorithm is based on comparative analysis (Case-Based/Data-Driven Diagnostics), which utilizes only the internal temperature from the sensor along with process and external data (weather, route, cargo), eliminating the need to mount an external temperature sensor on every container. The operation of the algorithm is illustrated by the flowchart in Figure 2.

The algorithm estimates the Thermal Integrity Index (*I_TI_*) based on the determined internal temperature increase profile ($\frac{\mathrm{dT}}{\mathrm{dt}}$), and then compares it with a historical baseline established for analogous transport conditions (the so-called Digital Twin/Similar Historical Cases).

**Figure 2:**
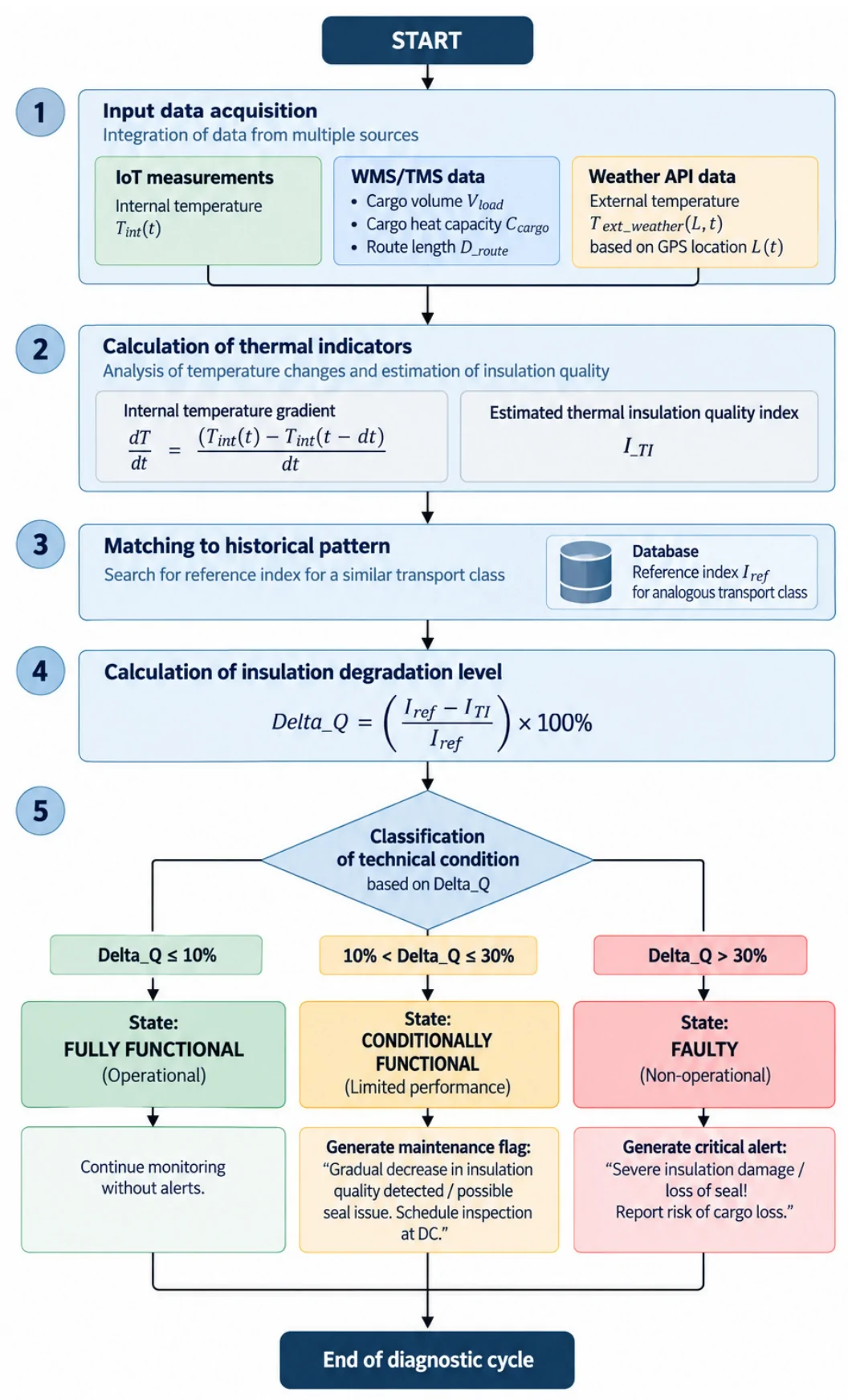
Block diagram of the algorithm operation.

The diagnostic framework operates on a store-and-forward and batch/on-demand analytical model rather than pure real-time streaming. Temperature and environmental data are initially logged and stored in the internal memory of the IoT sensors. Upon establishing a network connection (e.g., via gateways or mobile network during transit or upon return to the distribution center), the collected datasets are automatically transmitted and aggregated in the cloud repository. Consequently, the diagnostic algorithm presented in this study processes historical and logged datasets. It is executed cyclically (e.g., post-trip or at specific milestones) or on-demand by logistics operators. The algorithm retrieves the recorded internal temperature profile *T_int_(t)* alongside corresponding process data (WMS/TMS) and external weather API data (*T_ext_weather_)(L,t)*) corresponding to the specific transport window, enabling a comprehensive post hoc or periodic evaluation of thermal insulation integrity.

The algorithm utilizes the following measurement data (IoT):

*T_int_*(*t*)—continuous reading of the internal coolbox temperature.

Process and cargo data (WMS/TMS):

*V_load_*—cargo volume/fill rate of the container [%],

*C_cargo_*—specific heat capacity of the cargo (type of goods, e.g., meat, dairy, vegetables),

*D_route_*—planned route length/duration,

*L*(*t*) —approximate GPS location (allows mapping of the route stage).

External data (Weather API):

*T_ext_*___*_weather_*(*L*,*t*)—forecasted/historical ambient temperature from a weather service based on location *L(t)* and time *t*.

Description of the Algorithm’s Operation

Step 1: Determination of instantaneous and cumulative temperature gradients

The algorithm calculates the instantaneous rate of temperature change inside the container:

(1) $$\frac{dT_{\mathrm{int}}}{\mathrm{dt}}=\frac{T_{\mathrm{int}}(t)-T_{\mathrm{int}}(t-∆t)}{∆t}$$

Step 2: Determination of the Estimated Thermal Integrity Index (*I_TI_*)

The algorithm estimates the Thermal Integrity Index (*I_TI_*) based on the determined internal temperature increase profile ($\frac{dT_{\mathrm{int}}}{\mathrm{dt}}$). Rather than calculating absolute heat flow, *I_TI_* functions as a normalized dimensionless or scaled diagnostic indicator derived from the fundamental heat balance equation of the container system. Assuming a lumped capacitance approach for the internal payload and neglecting transient wall capacity compared to the internal thermal mass, the rate of heat infiltration through the insulated walls can be expressed via Fourier’s law of heat conduction combined with the container’s thermal characteristics:

(2) $$Q_{\mathrm{loss}}=U\cdot A\cdot \left(T_{\mathrm{ext}\_\mathrm{weater}}(L,t)-T_{\mathrm{int}}(t)\right)=\frac{k_{\mathrm{eff}}}{d}\cdot A\cdot \left(T_{\mathrm{ext}\_\mathrm{weather}}(L,t)-T_{\mathrm{int}}(t)\right)$$

where *U* is the overall heat transfer coefficient, *A* is the mean surface area of the coolbox, *k_eff_* is the effective thermal conductivity of the composite walls (PUR foam and PE casing), and *d* is the nominal wall thickness. Balancing this heat infiltration against the sensible heat absorbed by the cargo (defined by cargo mass *m_cargo_* and specific heat capacity *C_cargo_*):

(3) $$m_{\mathrm{cargo}}\cdot C_{\mathrm{cargo}}\cdot \frac{dT_{\mathrm{int}}}{\mathrm{dt}}=\frac{k_{\mathrm{eff}}\cdot A}{d}\left(T_{\mathrm{ext}\_\mathrm{weather}}(L,t)-T_{\mathrm{int}}(t)\right)$$

By rearranging this governing energy balance, the Estimated Thermal Integrity Index (*I_TI_*) is formulated to isolate the effective insulation-geometry ratio (*k_eff_/d* or proportional conductance factor) scaled by load parameters:

(4) $$I_{\mathrm{TI}}=\frac{\left(T_{\mathrm{ext}\_\mathrm{weater}}(L,t)-T_{\mathrm{int}}(t)\right)\cdot f\left(V_{\mathrm{load},}C_{\mathrm{cargo}}\right)}{\frac{dT_{\mathrm{int}}}{\mathrm{dt}}}$$

where *f* (*V_load_*, *C_cargo_*) represents a normalization function compensating for varying fill volumes and specific heat capacities of the transported goods. This formulation allows I_TI_ to serve as a consistent health indicator reflecting deviations from the nominal thermal resistance.

Step 3: Comparative Analysis with the Reference Transport Database (Historical Pattern Matching)

The algorithm searches the historical database for a transport class with similar parameters:

(5) $${\mathrm{Class}}_{\mathrm{ref}}=f\left(D_{\mathrm{route}},\;V_{\mathrm{load}},\;C_{\mathrm{cargo}},{\bar{T}}_{\mathrm{ext}\_\mathrm{weather}}\right)$$

For a given class, the historical average insulation quality value for a fully functional container is determined.

Step 4: Degradation Detection and Technical Condition Classification

The decrease in the technical insulation quality (Degradation Index) is calculated:

(6) $$∆Q=\frac{{\bar{I}}_{\mathrm{TI},\mathrm{ref}}-I_{\mathrm{TI}}(t)}{{\bar{I}}_{\mathrm{TI},\mathrm{ref}}}\cdot 100\%$$

Based on the result of the above function, the technical condition of the container is determined according to the following:

- ∆*Q* ≤ 10% FULLY FUNCTIONAL

The container maintains its thermal parameters in accordance with the historical baseline for this route and cargo.

- 10% < ∆*Q* ≤ 30% CONDITIONALLY FUNCTIONAL

Accelerated heat leakage is occurring due to micro-leaks, worn gaskets, or an improperly fastened latch. The container requires a technical inspection upon return to the DC.

- ∆*Q* > 30% FAULTY

Rapid drop in insulation performance (broken gasket, damaged PUR wall, door ajar). The system triggers an alarm and forecasts the risk of cargo spoilage along the route.

### 2.4. Evaluation of Method for Automatic Detecting Technical State of Coolbox

#### 2.4.1. Testing Laboratory

The experimental tests were carried out in the Laboratory of Techno-Climatic Research and Heavy Duty Machines (LBT) at Cracow University of Technology. The laboratory is accredited by the Polish Centre for Accreditation (PCA) and operates an advanced large-scale walk-in climatic chamber designed for environmental and thermal endurance testing of full-sized vehicles, containers, and industrial machinery. The laboratory is an accredited research facility holding Defense and Security (OiB) accreditation granted by the Minister of National Defence of Poland under accreditation scope No. 55/MON/2024. The laboratory’s scope of testing includes evaluating total resistance to low and high ambient temperatures according to NO-06-A107:2021 (points 4.2 and 4.3), NO-A-STANAG-4370/AECTP-300-2:2022, and NO-A-STANAG-4370/AECTP-300-3:2022. Additionally, it encompasses testing resistance to rapid ambient temperature changes at maximum rates attainable within the thermoclimatic chamber, pursuant to NO-06-A107:2021 (point 4.5.4), as well as resistance to condensed atmospheric precipitation such as frost and dew in accordance with NO-06-A107:2021 (point 4.10).

The climatic chamber provides a wide operational temperature range from −51 °C to +71 °C with precise temperature regulation (±0.5 °C) and controlled relative humidity (ranging from 10% to 98% RH for positive temperatures). Its large internal volume enables the simultaneous placement and testing of multiple insulated coolboxes alongside real-time data acquisition hardware under uniform environmental conditions. The controlled chamber environment ensured that external ambient fluctuations were eliminated during the baseline cooling, stabilization, and ambient exposure phases of the testing protocol.

#### 2.4.2. Scenario of Completed Tests

The scenario assumes creating (simulating) the exposure of coolboxes—in which two temperature and humidity sensors were mounted each—to various climatic conditions within designated time intervals. In the environment surrounding the coolboxes, two additional sensors were used to measure the ambient temperature. The data collection interval for each of the sensors used was set to 10 min. During the test, two Gateway-type devices were used to collect data from the sensors.

Figure 3 shows the arrangement of the coolboxes in the test chamber space along with the measuring sensors used and the sensors for measuring ambient temperature.

#### 2.4.3. Specification and Arrangement of Measuring Sensors

To verify the measurements taken by the sensors, Pt100-type resistance temperature detectors mounted in a four-wire measurement line configuration were additionally used during the testing:

- for measuring ambient temperature—8 units;
- for measuring the internal coolbox temperature—2 sensors inside each coolbox, totaling 8 sensors;
- for measuring the temperature of selected eutectic packs—3 sensors.

In each case, the sensors enabled the recording of the temperature distribution and, additionally, in the case of the ambient environment, the calculation of their average value. A detailed list of the sensors and detectors, along with a description of their locations, is provided in tabular form (Table 3)—including the measurement point number, sensor type, and a description of the measurement transmitter installation location—and in graphical form (Figure 4).

Figure 5 shows an example location of the ambient sensors, which were placed in front of the coolboxes situated in the central part of the chamber. The arrangement of the sensors inside the coolboxes is shown in Figure 6, Figure 7, Figure 8 and Figure 9.

The Pt100 sensors (T1 through T24) have a measurement uncertainty of 0.5 °C, while the SKK sensors (e.g., C23010) feature an accuracy of 0.5 °C with a measurement resolution (step) of 0.3 °C.

To evaluate different performance levels, the experiment utilized four separate units designated as coolboxes C1 through C4. A partially functional state was replicated in coolbox C1 by pulling the door closed up to the gasket and catching it on the latch’s pull-down pin, leaving the locking lever disengaged (Figure 10a). Coolbox C2 was configured to represent a malfunctioning state by leaving roughly a 15 mm gap when closing the door (Figure 10c). Baseline fully operational performance was achieved with coolbox C3, which was completely closed with its door lever fully locked. For coolbox C4, a segment of the seal was excised, and the door was secured identically to C3 (Figure 10b).

Under the experimental blueprint, three specific technical conditions were targeted for simulation: operational, conditionally operational, and faulty. Because four test containers were available, two units were dedicated to modeling the partially operational scenario.

#### 2.4.4. Outline Test Schedule

Table 4 presents the outline testing program for the sensors.

The tests were executed in accordance with the scenario. On the first day, ten sensors were prepared in the central part of the chamber, 8 of which were placed inside the coolboxes and two in their surroundings. Additionally, temperature transmitters were installed both in the surroundings and inside the coolboxes to verify the measurements. Furthermore, 8 eutectic packs were arranged between the setup coolboxes or on their top surface, on which three temperature sensors were mounted. Next, cooling to the transport temperature was carried out—in accordance with the scenario, +2 to +6 °C.

On the second day, a 2-h period of stable storage temperature for the coolboxes in the packing area was carried out from 8:00 a.m. to 10:00 a.m. At 10:00 a.m., after entering the chamber, two eutectic packs (+3 °C) were placed inside each coolbox, and the coolbox doors were closed. As a result, the 6-h storage process at normal temperature—specifically within the range of +10 to +25 °C—began.

Following the completion of the scenario execution, the coolboxes continued to be stored under the same conditions for approximately 17 h until Day 3, when the coolboxes were opened and all sensors were disassembled. At this stage, the execution of the scenario was concluded.

A graphical interpretation of the scenario execution is presented in Figure 11.

Figure 12 shows the temperature profiles recorded during the execution of the scenario. It should be noted that the eutectic packs used in these studies (“Fresh/Chilled” variant with a eutectic temperature of +3 °C) exhibited an initial temperature slightly above 3 °C, which subsequently rose further. This indicates that the measurement process captured only the steady-state phase, as no actual phase change (melting) of the eutectic packs took place during the tests.

Figure 12 illustrates the comparative internal temperature trajectories (*T_int_(t)*) recorded across the four tested coolboxes (C1 through C4) during the 6-h thermal exposure phase at standard ambient conditions (+10 °C to +25 °C chamber temperature). Quantitative analysis of the heating rates ($\frac{dT_{\mathrm{int}}}{\mathrm{dt}}$) reveals distinct thermal degradation profiles:

- Fully Functional Container (C3): Maintained the highest thermal inertia, exhibiting a mean internal temperature increase rate of approximately ∆*T*/∆*t* ≈ 0.18 °C/h during the stabilization phase.
- Damaged Seal Container (C4-missing seal section): Demonstrated a significantly accelerated internal heating rate of ∆*T*/∆*t* ≈ 0.29 °C/h under identical ambient thermal loads.

Comparing these gradients confirms that compromised perimeter sealing (as in C4) accelerates internal heat infiltration and heating rates by approximately 60% relative to the fully functional baseline (C3), validating the diagnostic sensitivity of the proposed index.

### 2.5. Algorithm Performance Evaluation on Experimental Data

To validate the practical performance and diagnostic efficacy of the proposed framework, the temperature profiles recorded during the climatic chamber trials (Figure 12) were processed through the analytical algorithm. Using the external ambient reference temperature (*T_ext_*___*_weather_* ≈ 17 °C) during the steady phase and the internal temperature gradients ($\frac{dT_{\mathrm{int}}}{\mathrm{dt}}$) extracted from sensors T03–T10, the Thermal Integrity Index (*I_TI_*) and degradation level (∆*Q*) were computed for each coolbox configuration relative to the fully functional baseline container (C3).

Table 5 summarizes the quantitative outputs generated by the algorithm across the tested coolbox states.

The results confirm that the algorithm successfully differentiates between operational states, accurately classifying minor mechanical irregularities (C1) within the conditional threshold (10% < ∆*Q* < 30%) and severe thermal leaks (C2, C4) well above the critical threshold (∆*Q* > 30%).

## 3. Discussion

The experimental findings demonstrate that micro-temperature dynamics inside passive insulated coolboxes serve as sensitive indicators of structural state degradation and material aging. Conventional industrial assessment of thermal insulation degradation—primarily caused by the microstructural breakdown of Polyurethane (PUR) foam, blowing gas diffusion, and mechanical fatigue of Polyethylene (PE) walls or rubber gaskets—relies heavily on destructive laboratory testing. Standard methods (such as extracting core samples to measure thermal conductivity according to ISO 8301 or EN 12667) are expensive, time-consuming, and cause irreversible damage to the container, permanently removing it from the logistics fleet.

In contrast, the data-driven framework introduced in this study offers a fully non-destructive diagnostic approach that seamlessly operates during real-world logistics operations. By shifting the evaluation methodology to a comparative pattern-matching model (Case-Based Diagnostics/Digital Twin), the technical condition of a coolbox is continuously monitored throughout its operational lifecycle without damaging its physical structure.

A primary operational advantage of the proposed method is the complete elimination of external temperature sensors mounted on individual coolboxes. Hardware deployed on container exteriors in retail and distribution environments is prone to severe mechanical damage, high-pressure water destruction during container washing cycles (at >60 °C), and theft. The present algorithm circumvents these hardware limitations by utilizing a single internal IoT temperature sensor. External ambient thermal exposure is dynamically retrieved via location-based weather APIs using approximate GPS coordinates from the transport management system. By linking internal heating rates with contextual parameters—specifically cargo volume, cargo heat capacity, and route duration—the algorithm isolates true structural insulation decay from external environmental influences.

However, a fundamental prerequisite for the practical deployment of this diagnostic model is operational repeatability. The algorithm relies on data accumulated over multiple transport cycles executed along similar routes under comparable logistics conditions. When coolboxes are repeatedly deployed on recurring distribution loops (e.g., daily DC-to-store deliveries), the algorithm establishes a reliable historical baseline. Sudden deviations from this baseline indicate immediate operational faults (e.g., unlatched locking levers or door gaps, as observed in C1 and C2), whereas gradual, persistent trends in thermal integrity decay over several months signify material aging and seal wear (as simulated in C4). This distinction enables logistics operators to transition from reactive repairs to predictive, condition-based maintenance, inspecting or re-sealing containers only when progressive thermal decay is detected, thereby extending asset lifespan while safeguarding food quality.

Limitations and Future Work:

Despite the promising diagnostic performance demonstrated during the controlled climatic chamber trials, several limitations of the current study should be explicitly acknowledged. The empirical validation presented in this work is based on a specific test scenario comprising four coolboxes evaluated under a controlled thermal profile. While this setup successfully validated the algorithmic differentiation between baseline, minor latching deviations, and severe seal/structural failures, real-world cold chain logistics involve a much wider spectrum of stochastic variables—such as highly fluctuating ambient weather conditions, multi-stage transport loops, varying cargo fill volumes, and prolonged operational aging over multiple years.

To ensure broader robustness, future research will focus on scaling the experimental validation across diverse seasonal weather profiles, extended fleet operations, and a larger population of passive containers. Furthermore, expanding the historical reference database through continuous data ingestion from commercial IoT deployments will further enhance the predictive accuracy and generalization capabilities of the proposed diagnostic framework.

## 4. Conclusions

This study introduced a novel, non-destructive diagnostic methodology for the continuous assessment of technical state decay and thermal insulation degradation in passive coolboxes. By capturing internal microclimatic trends via a single wireless IoT sensor and correlating them with ambient weather APIs and cargo data, the proposed framework successfully evaluates container integrity during active transit.

The empirical results and theoretical contributions of this work lead to the following key conclusions:

- Elimination of Destructive Testing: The proposed method replaces traditional, irreversible, and costly laboratory tests (such as core sample extraction for thermal conductivity measurements) with a fully non-invasive inline diagnostic tool. This allows logistics operators to track the gradual aging of PUR insulation foam and gasket wear throughout the container’s operational lifespan without taking it out of service.
- Sensor-Lean Architecture: The algorithm eliminates the need for external ambient temperature sensors on container walls-which are highly vulnerable to mechanical impacts and high-temperature washing cycles—by successfully integrating internal IoT readings with external weather API data based on route geolocation.
- Dependence on Operational Repeatability: The diagnostic accuracy relies on the container being deployed repeatedly across similar transport routes and operational profiles. This repeatability enables the generation of robust historical baselines against which insulation performance loss can be reliably measured.
- High Diagnostic Sensitivity: Experimental testing in an accredited thermoclimatic chamber confirmed that internal temperature gradients clearly differentiate between fully functional containers (C3), minor latching oversights (C1), gasket degradation (C4), and critical structural breaches (C2). Uncompressed or damaged seals accelerate internal heating rates by up to 60%, drastically reducing the passive cooling window.
- Predictive Cold-Chain Traceability: Integrating this data-driven model into warehouse management systems enables condition-based maintenance strategies. Containers exhibiting minor seal degradation can be flagged for gasket replacement at distribution centers before critical thermal thresholds are violated, thereby reducing food spoilage risks and ensuring ATP compliance.

## Figures and Tables

**Figure 3:**
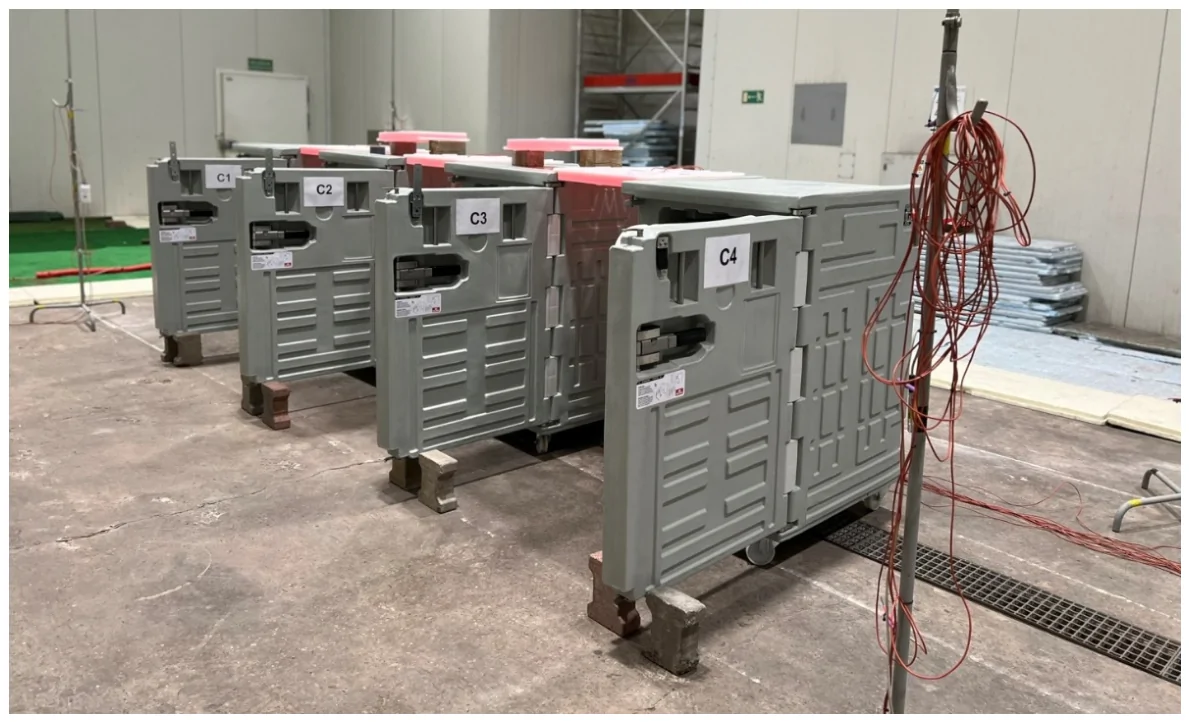
Arrangement of coolboxes before the start of the scenario execution (cooling to the “fresh” transport range).

**Figure 4:**
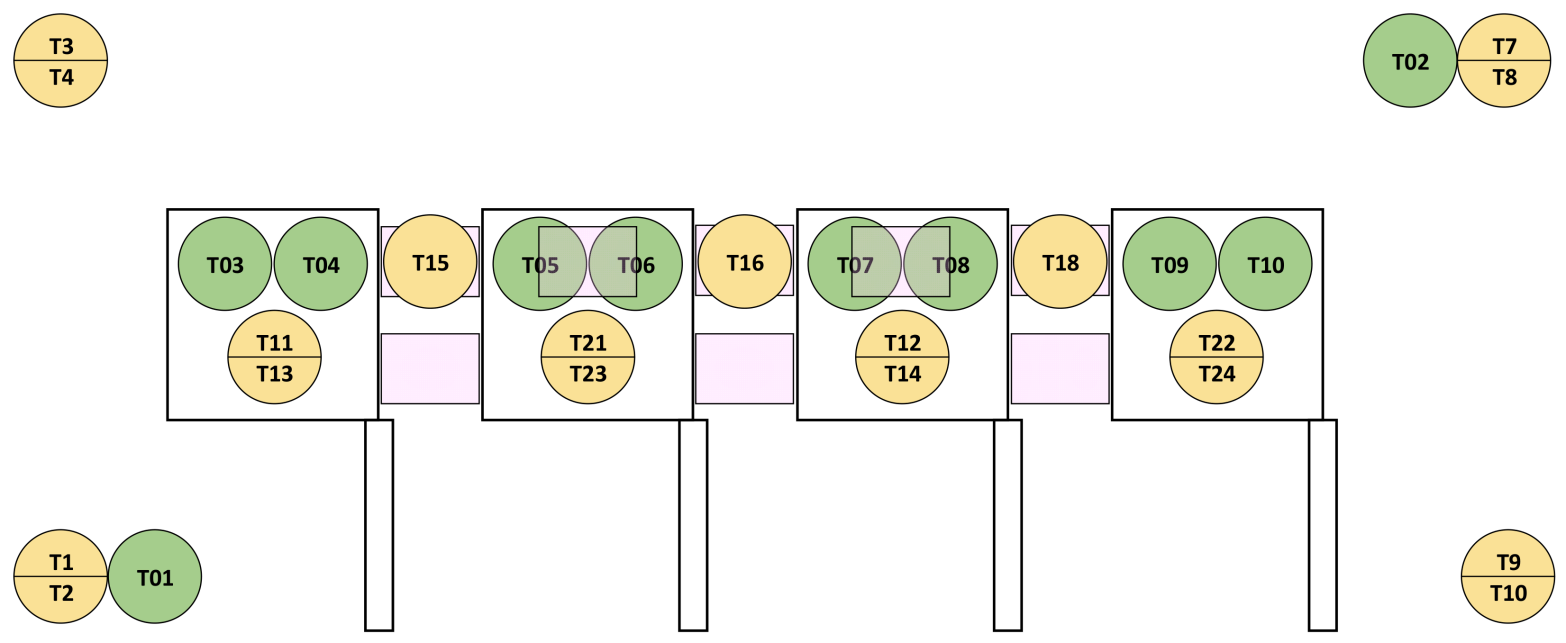
Arrangement of sensors (green color) and measurement transmitters (yellow color) against the background of open coolboxes (black color) and eutectic packs (pink color) during the cooling process.

**Figure 5:**
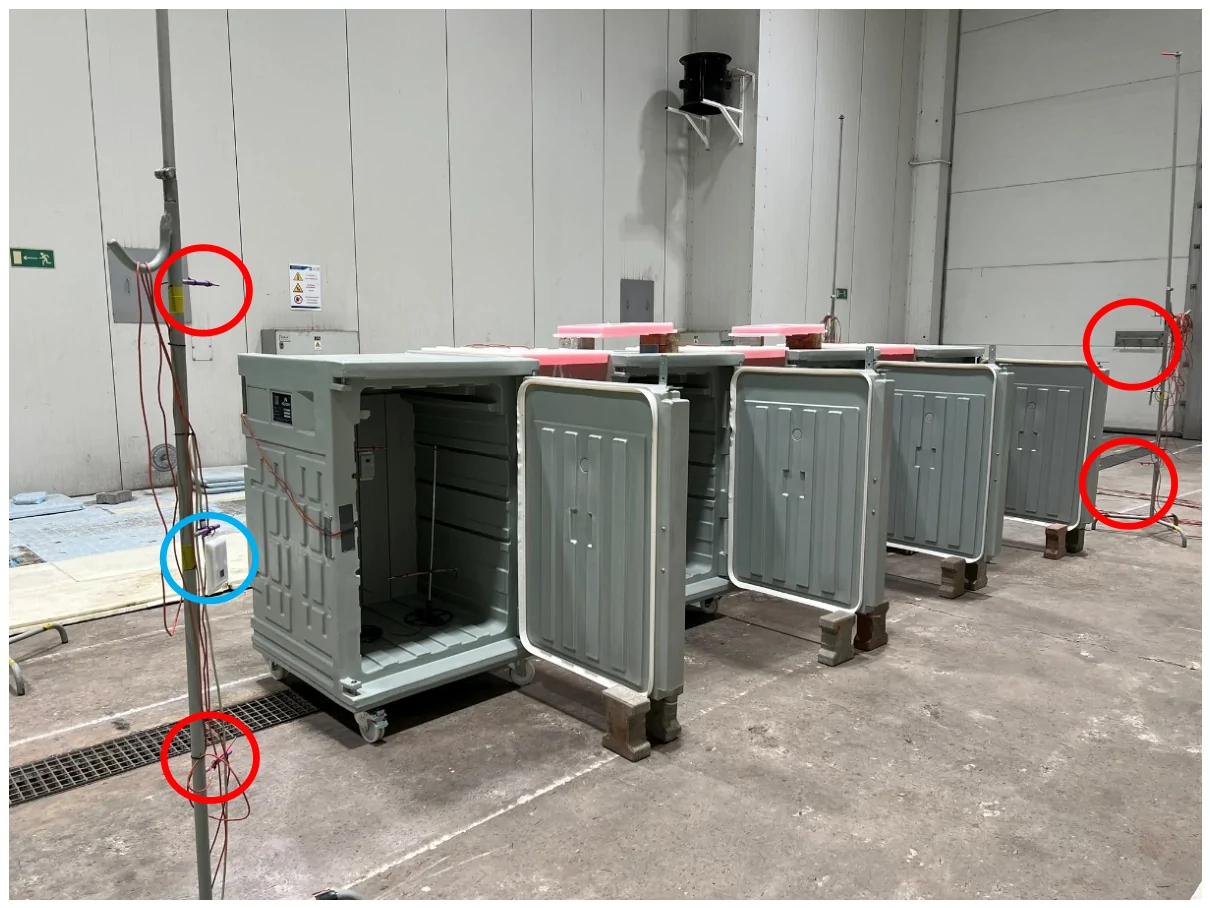
Example location of temperature sensors in the environment of the workstation used during the execution of the scenario.

**Figure 6:**
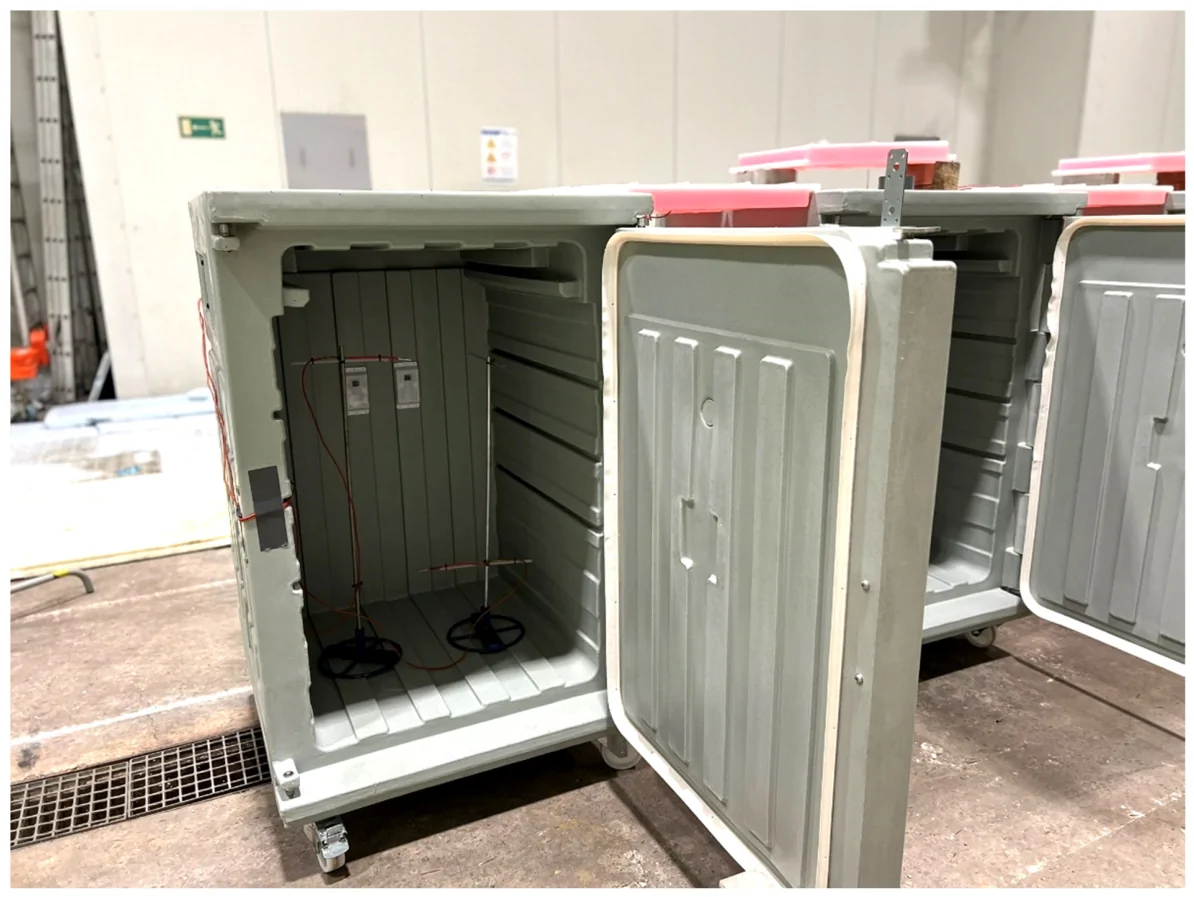
Location of T11 and T13 temperature sensors inside coolbox C1.

**Figure 7:**
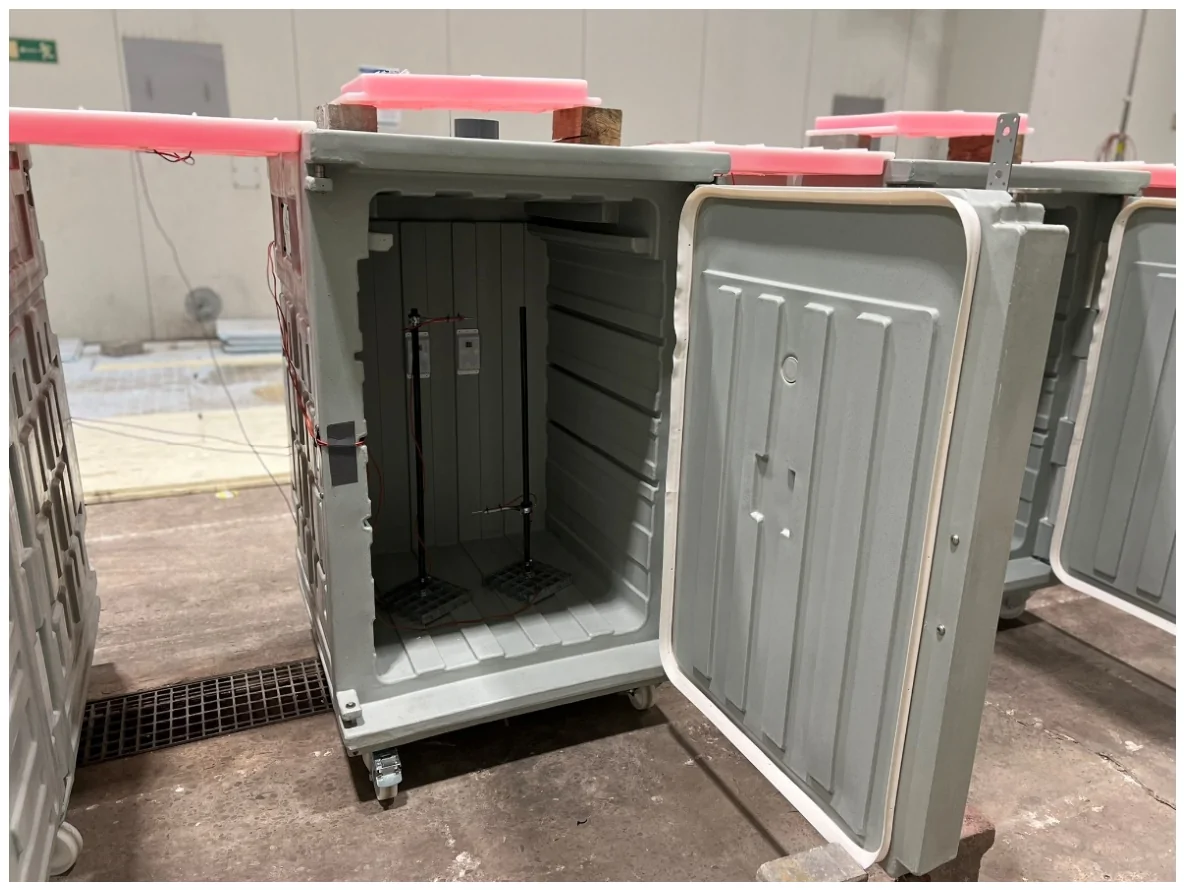
Location of T21 and T23 temperature sensors inside coolbox C2.

**Figure 8:**
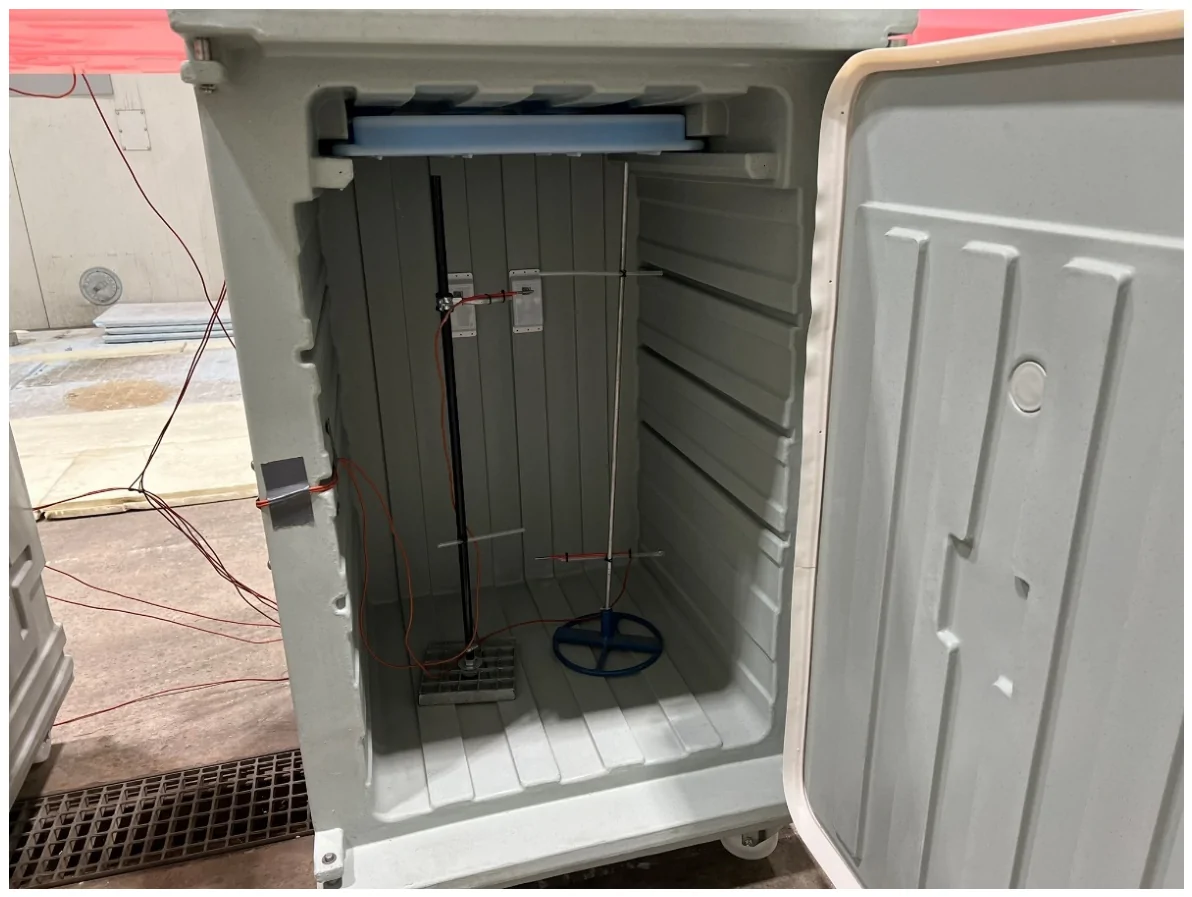
Location of T12 and T14 temperature sensors inside coolbox C3.

**Figure 9:**
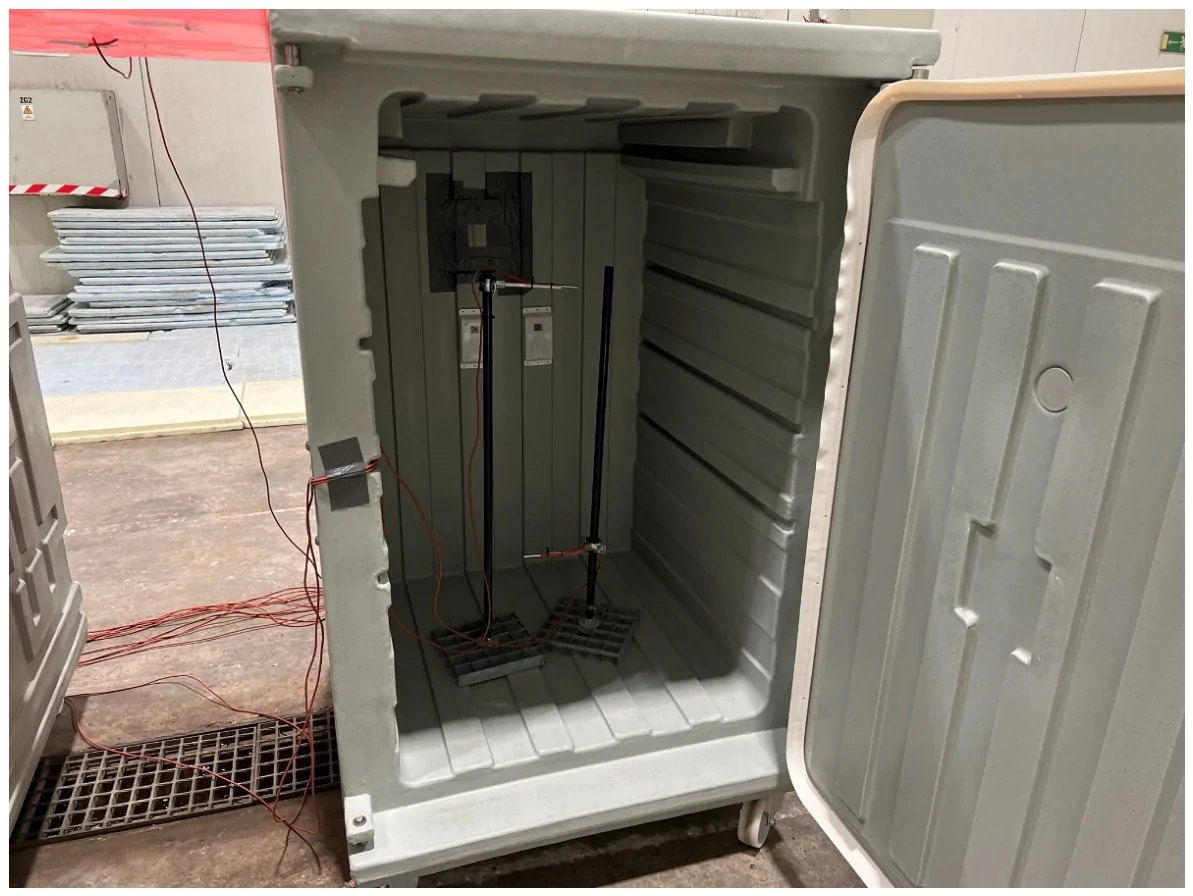
Location of T22 and T24 temperature sensors inside coolbox C4.

**Figure 10:**
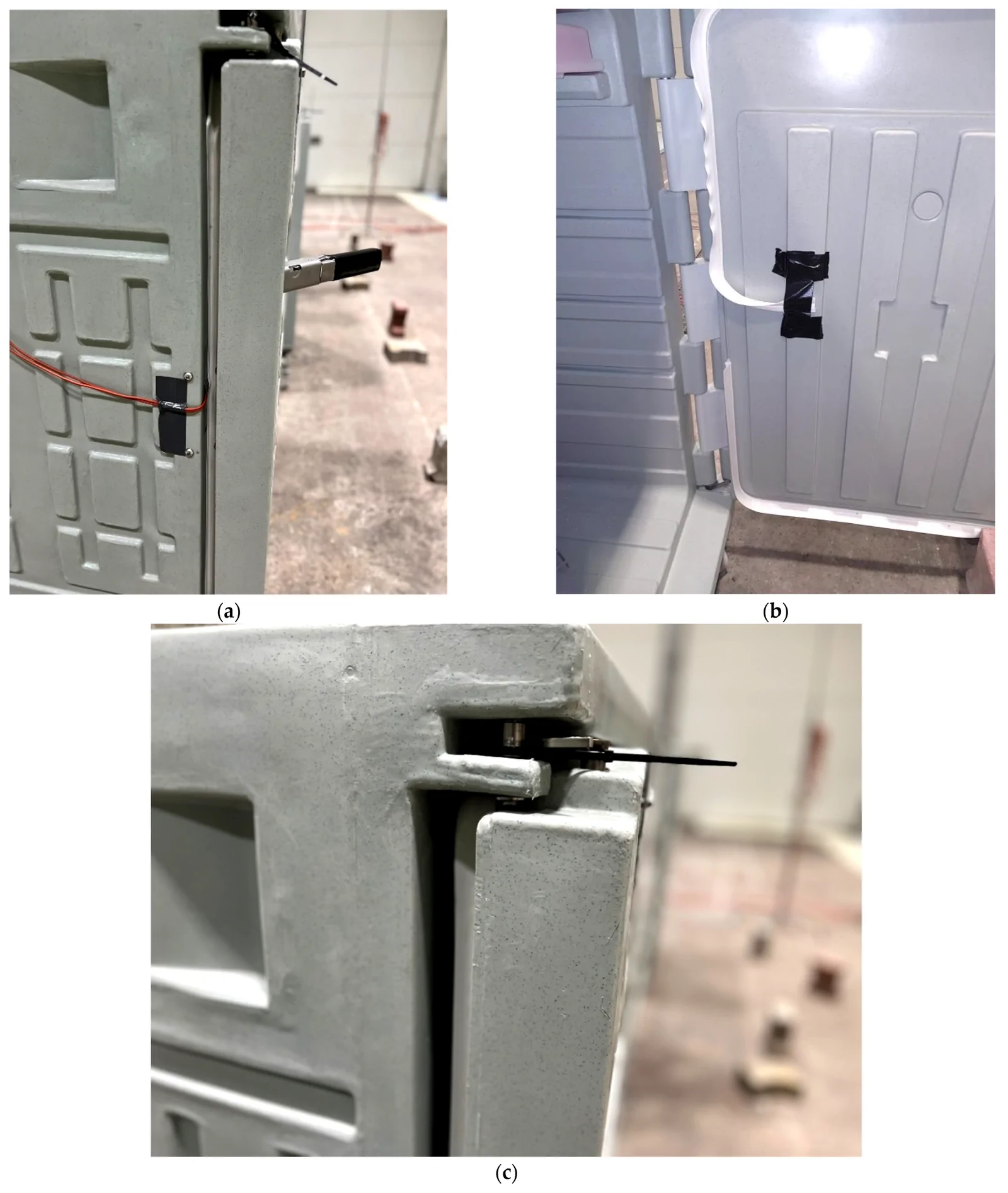
Method for simulating the technical states of individual coolboxes, without engaging the lever (**a**); the door closed in the identical manner (**b**); closing the door to a gap of approx. 15 mm (**c**).

**Figure 11:**
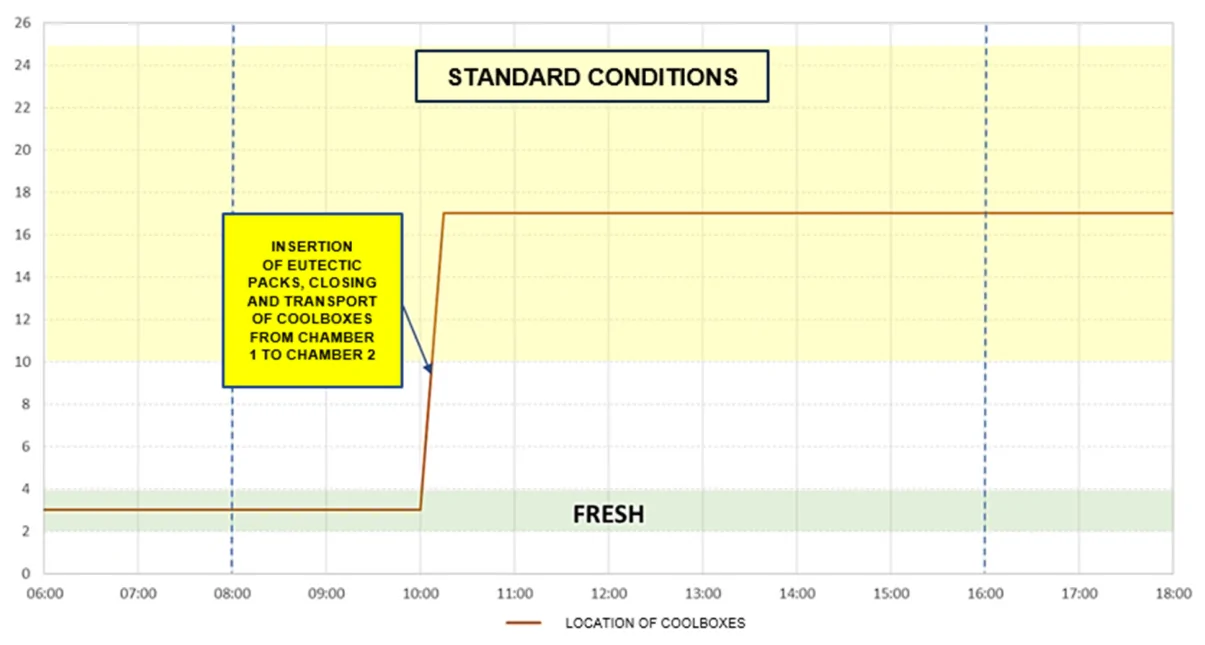
Graphical interpretation of the execution of the scenario.

**Figure 12:**
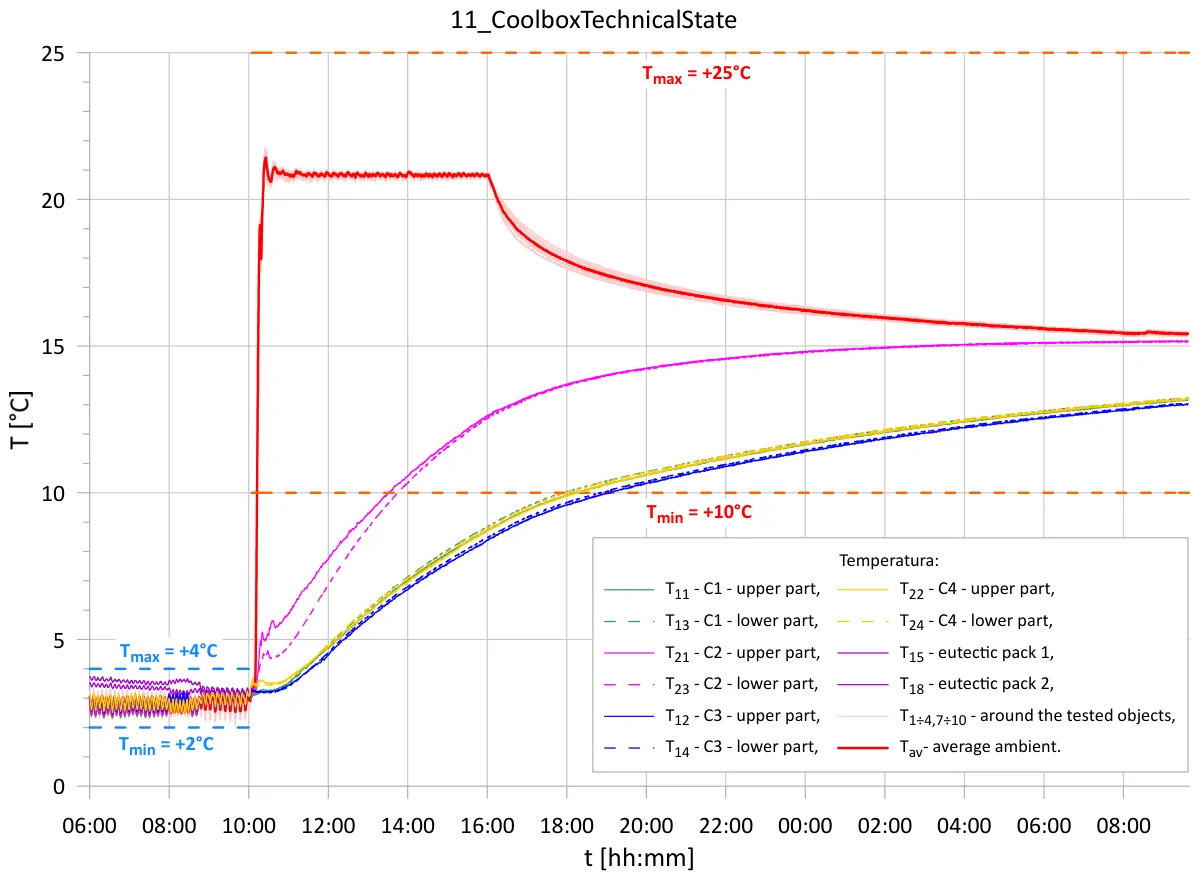
Temperature profiles recorded during the execution of the scenario.

**Table 2:** Logistics cycle for coolboxes [34].

Process	Obligatory	Zone	Scanning	Frequency per Cycle	Environmental Temperature of Coolbox	Notes
**Arrival at DC**	Yes	Receiving/Dispatch	Yes	1	2 °C ÷ +20 °C	
**Container Washing**	Yes	Receiving/Dispatch	No	In every cycle or every few cycles	60 °C	High-pressure cleaning, chemicals, high temperature > 60 °C
**Drying**	No	Cleaning Zone/Storage Zone CB	Recommended	As often as washing	2 °C ÷ +50 °C	Natural drying or with forced air circulation and higher temperature
**Cooling**	Recommended	Receiving/Dispatch	Recommended-in the absence of an automatic system	Always	−20 °C ÷ +6 °C	Cooling to a specified temperature (Fresh or Frozen)
**Loading of Products/Goods**	Yes	Drying Zone/Storage Zone CB	Recommended	1	2 °C ÷ +6 °C	In the Fresh zone
**Placement of the coolant**	Yes	Storage	No	1	2 °C ÷ +6 °C	Loading dry ice or eutectic coolant
**Coolbox transit to the gate**	Yes	Receiving/Dispatch	Recommended	1	2 °C ÷ +6 °C	
**Loading onto the trailer**	Yes	Receiving/Dispatch	Yes	1	2 °C ÷ +6 °C	
**Transport to the store**	Yes	Receiving/Dispatch	Yes	1	Atmospheric	Temperature depending on the season
**Delivery to the store**	Yes	Transport	Yes			
**Unloading of the coolbox at the store**	Yes	Transport	No	1	Atmospheric or fresh	Duration usually ranges from a few minutes to several hours from delivery
**Waiting for transport**	Yes	Shop	No	1	Atmospheric	During the next delivery, approximately 24 h.
**Loading onto the trailer**	Yes	Shop	Yes	1	Atmospheric	
**Transport to the DC**	Yes	Transport	No	1	Atmospheric	

**Table 3:** List of locations of sensors and measurement transmitters used during the testing.

Measurement Point No.	Sensor Type	Description of Sensor/Measurement Transmitter Installation Location
T01	C23010540	Ambient, front-left of coolboxes, at a height of 1 m
T02	C23010713	Ambient, rear-right of coolboxes, at a height of 1 m
T03	C23010369	Inside coolboxC1, left
T04	C23010454	Inside coolboxC1, right
T05	C23010518	Inside coolboxC2, left
T06	C23010481	Inside coolboxC2, right
T07	C23010522	Inside coolboxC3, left
T08	C23010462	Inside coolboxC3, right
T09	C23010383	Inside coolboxC4, left
T10	C23010372	Inside coolboxC4, right
T1	Pt100,Temp: [°C]	Ambient, top front-left of the setup, at a height of 1.5 m from the ground
T2	Pt100,Temp: [°C]	Ambient, bottom front-left of the setup, at a height of 0.5 m from the ground
T3	Pt100,Temp: [°C]	Ambient, top rear-left of the setup, at a height of 1.5 m from the ground
T4	Pt100,Temp: [°C]	Ambient, bottom rear-left of the setup, at a height of 0.5 m from the ground
T7	Pt100,Temp: [°C]	Ambient, top rear-left of the setup, at a height of 1.5 m from the ground
T8	Pt100,Temp: [°C]	Ambient, bottom rear-left of the setup, at a height of 0.5 m from the ground
T9	Pt100,Temp: [°C]	Ambient, top front-left of the setup, at a height of 1.5 m from the ground
T10	Pt100,Temp: [°C]	Ambient, bottom front-left of the setup, at a height of 0.5 m from the ground
T11	Pt100,Temp: [°C]	Coolbox 1, top at a height of 0.7 m from the coolbox floor
T12	Pt100,Temp: [°C]	Coolbox 3, top at a height of 0.7 m from the coolbox floor
T13	Pt100,Temp: [°C]	Coolbox 1, bottom at a height of 0.2 m from the coolbox floor
T14	Pt100,Temp: [°C]	Coolbox 3, bottom at a height of 0.2 m from the coolbox floor
T15	Pt100,Temp: [°C]	Eutectic pack, between C1 and C2, attached to the bottom
T16	Pt100,Temp: [°C]	Eutectic pack, between C2 and C3, placed loosely on top of the pack
T18	Pt100,Temp: [°C]	Eutectic pack, between C3 and C4, attached to the bottom
T21	Pt100,Temp: [°C]	Coolbox 2, top at a height of 0.7 m from the coolbox floor
T22	Pt100,Temp: [°C]	Coolbox 4, top at a height of 0.7 m from the coolbox floor
T23	Pt100,Temp: [°C]	Coolbox 2, bottom at a height of 0.2 m from the coolbox floor
T24	Pt100,Temp: [°C]	Coolbox 4, bottom at a height of 0.2 m from the coolbox floor

**Table 4:** Outline test program according to the scenario.

No.	Detailed List of Tasks to Be Executed	Timeframe
1.	Placement of sensors at the workstation, installation of measuring detectors	Day 1
2.	Commencement of the cooling process for coolboxes and eutectic packs to the “fresh” transport temperature	
3.	Reaching the required temperature, commencement of the stabilization process	
4.	Placement of eutectic packs inside the coolboxes and closing them	Day 2
5.	Commencement of the chamber heating process to normal temperature, simultaneously initiating a 6-h storage period at this temperature	
6.	Storage of containers at this temperature for a period longer than specified in the test schedule (17 h)	Day 3
7.	Opening of the coolboxes and disassembly of temperature sensors	

**Table 5:** Diagnostic algorithm results and technical state classification for experimental coolboxes.

Coolbox ID	Simulated Technical State/Condition	Thermal Integrity Index (ITI) [h °C]	Degradation Index (ΔQ) [%]	Algorithm Classification and Action Flag
C3	Fully functional (Reference baseline, closed with lever)	48.5	0.0%	Fully functional (No alerts, continue monitoring)
C1	Conditionally functional (Latch engaged without lever)	38.2	21.2%	CONDITIONALLY FUNCTIONAL (Maintenance flag: potential seal/latch issue)
C4	Faulty (Section of perimeter seal removed)	29.1	40.0%	Faulty (Critical alert: severe insulation/seal breach)
C2	Faulty (15 mm door gap/unlatched)	24.0	50.5%	Faulty (Critical alert: high heat infiltration)

## Data Availability

The original contributions presented in the study are included in the article, further inquiries can be directed to the corresponding author.
